# Extraction of Cellulose from *Ulva lactuca* Algae and Its Use for Membrane Synthesis

**DOI:** 10.3390/polym15244673

**Published:** 2023-12-11

**Authors:** Claudia Ana Maria Patrichi, Doinita Roxana Cioroiu Tirpan, Ali A. Abbas Aljanabi, Bogdan Trica, Ioana Catalina Gifu, Tanase Dobre

**Affiliations:** 1 Chemical and Biochemical Engineering Department, University Politehnica of Bucharest, 1-6 Gheorghe Polizu, 011061 Bucharest, Romania; trica.bogdan@gmail.com (B.T.); gifu_ioanacatalina@yahoo.com (I.C.G.); tghdobre@gmail.com (T.D.); 2 Chemistry and Chemical Engineering Department, Ovidius University of Constanta,124 Mamaia Street, 900527 Constanta, Romania; 3 Al Mussaib Technical College, Al-Furat Al-Awsat Technical University, Babylon P.O. Box 51006, Iraq; dr.ali.aljanabi@atu.edu.iq; 4 National Research & Development Institute for Chemistry and Petrochemistry, ICECHIM, Splaiul Independentei No. 202, 6th District, 060021 Bucharest, Romania

**Keywords:** swelling, membrane, cellulose, algae, diffusion of swelling layer

## Abstract

Green algae are a sustainable source of biopolymers for the global demand due to their high photosynthetic efficiency. This article describes the extraction of cellulose from plant systems represented by *Ulva lactuca* species. In order to extract various substances, algae were finely ground with the help of solvents (liquid media). This was carried out to achieve the necessary conditions that help reduce the resistance this phase shows in regard to the transport and transfer of the species being extracted. The highest yield of extracted cellulose (20,944%) was obtained for the following factors: S/L = 1/20; conc. ethanol = 90%, conc. salts = 4 g/L. Hydrogel membranes are a unique class of macromolecular networks that contain a large fraction of aqueous solvent within their structure. With the cellulose extracted from algae, we obtained membranes which underwent the process of swelling in liquid media (ethyl alcohol) of different concentrations. The swelling of biocellulose membranes in alcoholic solutions of high concentrations was investigated. It was observed that the process of absorption of the alcoholic solution by the membrane occurred rapidly in the first part. After stabilization, the membranes continued to absorb at a slower rate until stabilization or saturation concentration was reached.

## 1. Introduction

In present, synthetic polymers began to widen their fields of use and they are find practically in all branches of the economy due to their excellent mechanical properties and competitive price. There are no limits regarding the use of polymer materials in the age of advanced technologies. Nevertheless, the particularity of these synthetic polymers obtained from monomers is their non-biodegradable behavior that affects the quality of the environment. Therefore, scientists focused their attention to biodegradable biopolymers, a current field of research with great ecological and economic importance. Due to their structure, biopolymers have unique properties that can be adapted for different uses [1,2].

Organisms like bacteria, plants and animals produce through biosynthesis biopolymers which could be separated by chemical or enzymatic treatment [3,4].

In recent decades, chemically synthesized fibers have been replaced by a wide range of biopolymers leading to the development of the bio-composite materials industry [5,6,7,8,9,10,11].

Seaweed represents a sustainable feedstock for the global demand of biopolymers. In nature, algae produce ten times more organic matter and release five times more oxygen than the entire terrestrial vegetation. Depending on the color of the predominant pigment, algae are classified into green, blue, red and brown. The waters of the seas and oceans carry huge amounts of algae of amazing diversity, organisms with a high growth rate.

*Ulva lactuca* (Phylum *Chlorophyta*, Class *Ulvophyaceae*, Order *Ulvales*, Family *Ulvaceae*) is a green alga which can be found on large areas off the Black Sea but also in coastal areas [12,13,14]. 

According to the study carried out by the Grigore Antipa Institute [15], for Romanian Black Sea *Ulva*, the biochemical composition of the algal powder of the *Ulva lactuca* sp. is the following: carbohydrates (54.95 ± 1.43%), mineral substances (24 ± 8.25%), proteins (14.58 ± 1.30%) and lipids (0.69 ± 0.06%). According to another study carried out by Lisha V.S. et al. [16], the seaweeds contain metals like zinc (28–64 mg.Kg^−1^), selenium (0–0.05 mg.Kg^−1^), manganese (45–454 mg.Kg^−1^), iron (18–102 mg.Kg^−1^), copper (18–102 mg.Kg^−1^), sulfur (0.4–1.4%), sodium (0.8–2.7%), potassium (0.7–2.4%), magnesium (0.3–0.7%) and calcium (0.1–3.0%). Comparatively, following other experimental studies, polysaccharides in *Ulva lactuca* are reported to comprise up to 45% [17], but it depends on the climatic region and the season of harvesting.

Polysaccharides are found mainly in the cell wall with an important role in the structural reinforcement of the algae. They are also present in the intercellular space in small proportions [18]. In *Ulva lactuca*, we can find two major polysaccharides, ulvans (made-up mainly of rhamnose, glucuronic acid, xylose, glucose and sulfate [19,20]) and cellulose, and two minors, xyloglucan and glucuronan. In comparison with xyloglucan, glucuronan and some the ulvans which are hydro soluble, cellulose is insoluble and can be separated from the other polysaccharides [21]. Currently, cellulose is one of the most commonly used substrates and has a wide range of uses, from the textile industry, tissue engineering, the biomedical industry and pharmaceuticals to the food industry and wastewater treatment [12,22,23]. Separation of cellulose from *Ulva lactuca* is easier to perform in the absence of lignin because there is no delignification in the separation scheme.

Hydrogels are a unique class of macromolecular networks that contain a large fraction of aqueous solvent within their structure.

The hydrophilicity of the network is due to the presence of chemical residues such as hydroxylic (-OH), carboxylic (-COOH), amidic (-CONH-), primary amidic (-CONH_2_), sulfonic (-SO_3_H) acids and others that can be found within the polymer backbone or as lateral chains. Nevertheless, it is also possible to produce hydrogels containing a significant portion of hydrophobic polymers, by blending or copolymerizing hydrophilic and hydrophobic polymers.

The hydrophilic/hydrophobic balance of the hydrogels, the degree of cross-linking and, especially, the degree of ionization and its interaction with counterions are the important parameters which control the equilibrium swelling, dimensional change and the release patterns of drugs from these carriers [24,25]. Hence, mathematical modeling of hydrogel swelling and predictability of swelling behavior has gained considerable attention during recent decades.

The first part of the article describes the extraction of cellulose from plants represented by algae. In order to extract various substances from algae, with the help of solvents, they are finely ground. This is performed in order to achieve the necessary conditions to reduce the resistance of this phase in terms of transport and transfer of extracted species. Considering these aspects, it was decided to extract cellulose from the powder obtained by grinding dried *Ulva lactuca* algae.

The second part of the article focuses on the conversion of obtained cellulose to membrane and the study of its use in removing water from solvent/water mixtures, including ethanolic solutions. Thus, from the extracted cellulose membranes were obtained that were tested for use as pervaporation membranes. The first step consisted of investigating the swelling in ethanolic solutions. The work has three elements of originality: (i) a proper procedure for cellulose extraction from *Ulva lactuca* algal species, abundant in the Black Sea; (ii) a solution for obtaining membranes starting from the obtained cellulose, enabling ethanolic solution pervaporation and (iii) experimental and modeling investigation with a new model of obtained membranes swelling in concentrated ethanolic solutions.

## 2. Materials and Methods

### 2.1. Materials

For cellulose extraction from algae, *Ulva lactuca* sp., ethyl alcohol (C_2_H_6_O 90%, 60%), sodium chloride (NaCl), ammonium oxalate ((NH_4_)_2_C_2_O_4_ 0.05%), acetic acid (CH_3_COOH 5%), sodium hypochlorite (NaClO 5%), sodium hydroxide (NaOH 0.5 M) and hydrochloric acid (HCl 5%) were used.

With respect to *Ulva Lactuca* algae composition, referring to the dry material, the literature data [26] also tested by us show that it contains moisture (0.12 ± 0.08), ash (0.18 ± 0.04), total nitrogen (0.022 ± 0.0019), proteins (0.145 ± 0.012), lipids (0.07 ± 0.005), carbohydrates (0.563 ± 0.062) and insoluble fibers (0.263 ± 0.05).

Sodium hydroxide (NaOH 10% VWR Chemicals), thiourea (CH_4_N_2_S 5% Merck, Bucharest, Romania), formaldehyde (CH_2_O 37% Merck, Bucharest, Romania) and ethyl alcohol (C_2_H_6_O 99%, 80%, 60%, 40%) were used to obtain membranes and to subject them to the swelling process.

### 2.2. Cellulose Extraction from Algae

**Figure 1:**
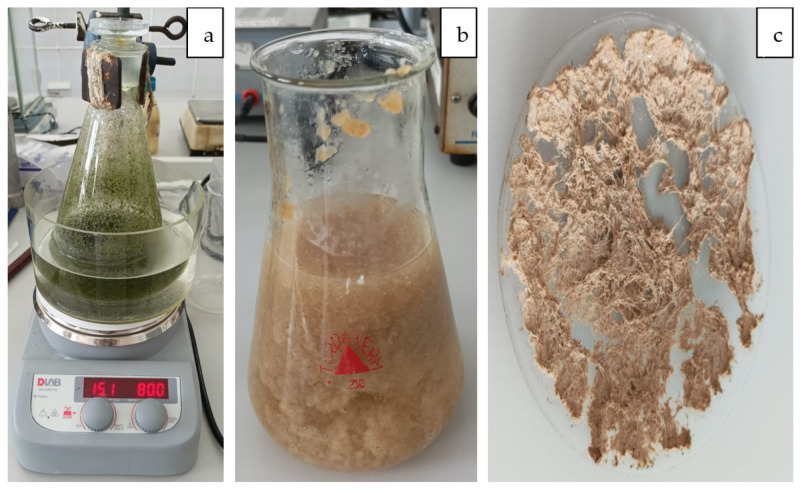
Stages of extracting cellulose from algae ((**a**) extraction, (**b**) washing, (**c**) drying).

The species *Ulva lactuca* sp. was harvested fresh from seawater, washed with distilled water and cleaned of impurities [27]. The algae were placed for 48 h in an apparatus (fruit dryer) with 5 overlapping trays, where they dehydrated evenly. The temperature of the warm air flow circulating horizontally was set to 50 °C in order to not destroy the bioactive substances found in algae [28,29]. Subsequently, the dried algae were finely ground by mortaring and subjected to observation by transmission (Figure 1) under an IOR ML-4M optical microscope (60-fold magnification (eyepiece 10 × 60)).

To extracted cellulose from algae species *Ulva lactuca*, 10 g of algae was introduced into a solution of ethyl alcohol [30]. The extraction was performed in a water bath with weak magnetic agitation (800 rot/min) at 65 °C in an Erlenmeyer flask with a ground glass stopper for 48 h (Figure 1a). The extractant was an ethanol/water mixture of various concentrations (90% and 60%, respectively). NaCl was also added in various concentrations 2 g/L and 4 g/L [5,27].

The ulvans were then extracted by adding 100 mL ammonium oxalate (0.05%) to the skimmed algae. The mixture was then heated for one hour between 90 and 100 °C while stirring. The residues of the hydrophilic fraction were removed by successive washings with hot water. The algae were bleached at 60 °C in a mixture of 200 mL acetic acid (5%) and 100 mL NaClO (5%) allowing removal of pigments (chlorophyll) (Figure 1b). Numerous extraction methods have been reported regarding conventional and unconventional procedures to separate chlorophyll. These can be intensified when using heating and/or stirring during extraction [31].

The algae were then washed with distilled water and dried at 105 °C. In the final extraction step, algae were treated with 100 mL NaOH (0.5 M) overnight at 60 °C under constant stirring conditions. The insoluble fraction was then washed to neutrality and dried. The powder obtained was mixed with 200 mL hydrochloric acid (5%), the mixture was heated to a boil, then the solution was left overnight at 30 °C while stirring. The extracted cellulose was washed to neutral pH and dried at 105 °C (Figure 1c).

### 2.3. Obtaining Membranes from Cellulose Extracted from Algae and the Process of Swelling

**Figure 2:**
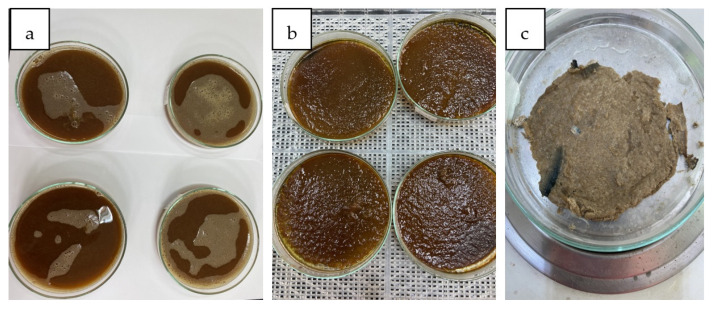
Stages of obtaining membranes from cellulose extracted from algae ((**a**) phase inversion, (**b**) crude membrane, (**c**) dried membrane).

In order to obtain the extracted cellulose membranes and to swell them, the following steps were followed. A solution of 150 mL containing 10% *w*/*w* NaOH and 5% of thiourea was prepared. In the alkaline solution, cellulose powder from algae was added at a concentration level between 7 and 10% by mass. The systems were kept under controlled agitation conditions and at a temperature of no more than 30 °C for a time of three hours. The slurry of cellulose in the alkaline solution was cooled at −10 °C for a period of 24 h. After observing the formation of cellulose gel in the alkaline solution, the cellulose gel was left to heat at room temperature (Figure 2a). We prepared 250 mL of slow acid solution with formaldehyde with which we washed the obtained membrane structure. We continued washing the membrane structure with distilled water until neutral pH was obtained (Figure 2b). The washed membranes were kept in distilled water with small formaldehyde concentration for a period of 24 h. The membranes were dried by water evaporation in a dehydrator at 70 °C for a period of 2 h, and after they were dried in a thermobalance at 100 °C (Figure 2c).

The membranes thus obtained underwent the process of swelling in an alcoholic solution. Ethyl alcohol of 99%, 80%, 60% and 40% concentration was used, and the mass of the membranes and the swelling time were recorded. The membrane was completely immersed in a sufficiently large volume of ethanolic solution. At certain intervals, the membrane was extracted, and we observed the increase in volume expressed quantitatively by the increase in its mass. This increase in mass is due to absorption from the water swelling medium.

## 3. Results and Discussion

### 3.1. Cellulose Extraction from Algae

**Figure 3:**
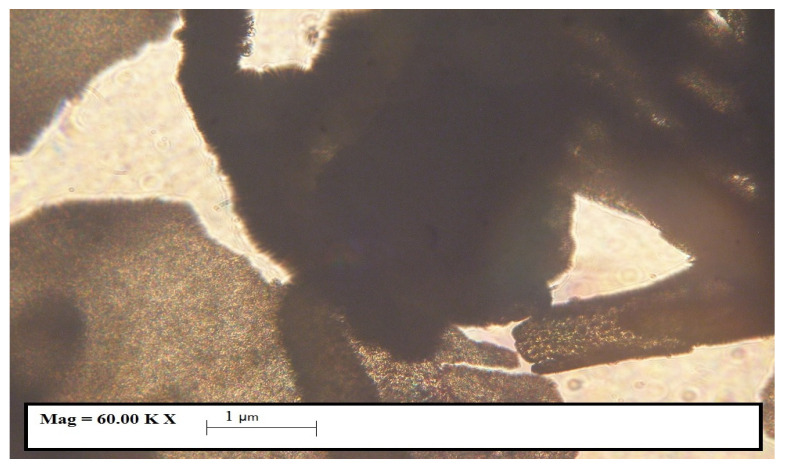
Microscopic image (60× magnification) of dry *Ulva lactuca* species.

The dried algae were subjected to observation by transmission (Figure 3) under the IOR ML-4M optical microscope (60-fold magnification (eyepiece 10 × 60)).

In Figure 3, it can be observed that the powder contains particles below 500 microns.

**Figure 4:**
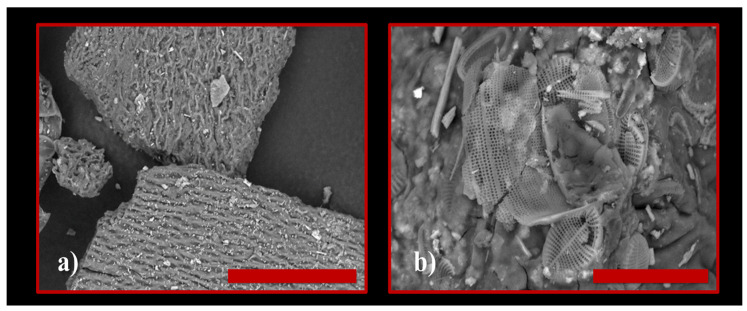
SEM image of dry *Ulva lactuca* species. Scale bar length: (**a**) 100 μm; (**b**) 30 μm.

Using the TM4000 Tabletop Scanning Electron Microscope (Chiyoda, Hitachi, Japan) we were able to analyze the powder of the species *Ulva lactuca* (Figure 4a,b).

In Figure 4a,b, we can very clearly observe microfibers (even nanofibers) but also certain cellulose structures.

In order to highlight the functional groups of species in the *Ulva lactuca* algae sample, Tensor 37 Bruker (Elmsford, Woodstock, NY, USA) equipment was used for FT-IR analyses.

**Figure 5:**
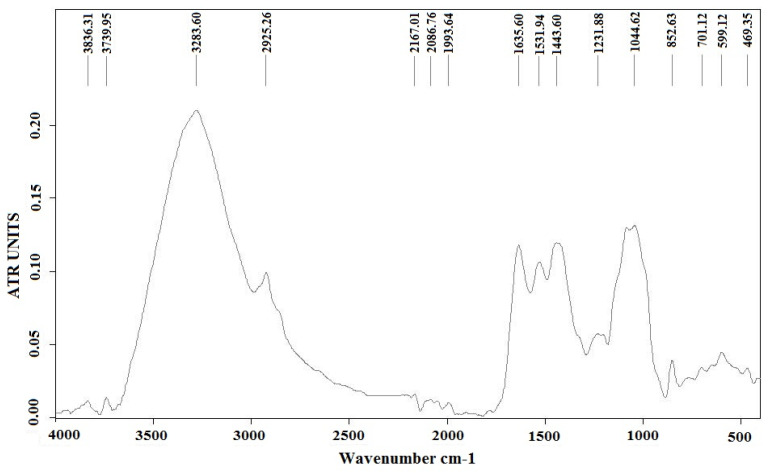
FT-IR spectrum of algal powder from species *Ulva lactuca* before the extraction.

The FTIR spectra in the region 4000–400 cm^−1^ for *Ulva lactuca* species powder is shown in Figure 5. The large width of the band at 3283.60 cm^−1^ indicates the presence of functional group -OH. The lipids from the green algae structure were identified using the band at 2925.26 cm^−1^. A broad band in the 1800–1500 cm^−1^ range with a wavelength of 1635.60 cm^−1^ can include proteins and amides I from the protein structure. Also, the wavelength 1531.94 cm^−1^ indicates the presence of amides II in the protein structure. The peak at 1231.88 cm^−1^ shows the carboxylic group COO^−^ and the characteristic band for phosphate P=O –esters. The presence of carboxylic acid and the –OH group is an indication of the presence of polysaccharides (especially cellulose), which is also confirmed by the peak at 1044.62 cm^−1^.

The extraction of cellulose was performed according to an experimental plan considering the following factors: solid/liquid ratio, ethanol concentration at extraction and concentration of salts in extractant; see Table 1.

The yield of extracted cellulose (R) is considered a process-dependent variable. The concentration of salts in the extractant was determined with a digital dissolved solids concentration analyzer (ppm). The highest yield of extracted cellulose (20,944%) was obtained for the following factors: S/L = 1/20; conc. ethanol = 90%, conc. salts = 4 g/L.

### 3.2. Dissemination of Membrane Precipitation Results and Membrane Swelling Process

**Figure 6:**
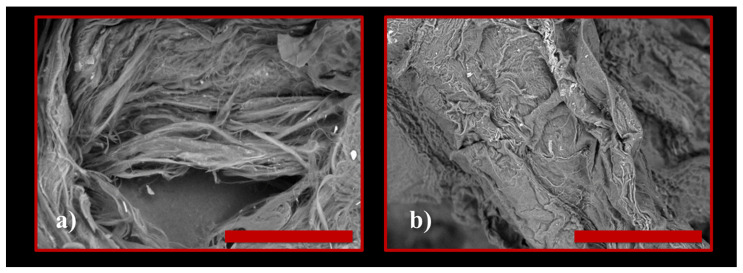
SEM image of cellulose from dry Ulva lactuca species. Scale bar length: (**a**) 50 μm; (**b**) 100 μm.

**Figure 7:**
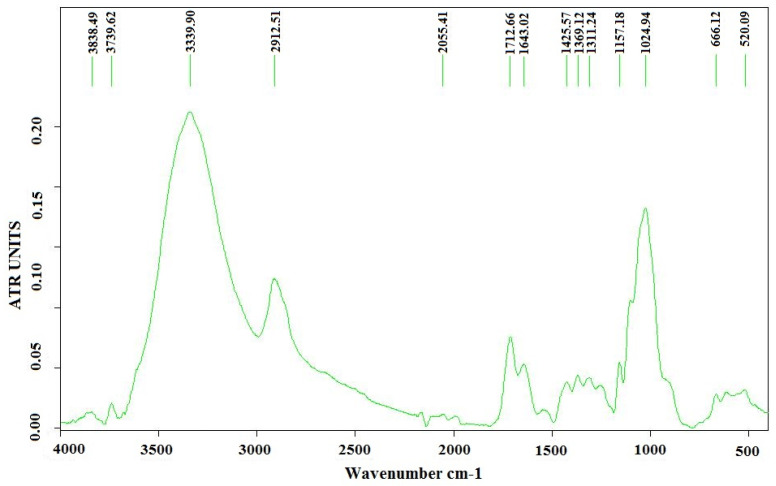
FT-IR analysis of the cellulose extracted from *Ulva lactuca* species.

Using the TM4000 Tabletop Scanning Electron Microscope (Chiyoda, Hitachi, Japan) we analyzed cellulose obtained from the species *Ulva lactuca* (Figure 6a,b). In order to highlight the functional groups of species in the *Ulva lactuca* algae sample, Tensor 37 Bruker (Elmsford, Woodstock, NY, USA) equipment was used for FT-IR analyses (Figure 7).

The spectral analysis of the cellulose extracted from *Ulva lactuca* species is presented in Figure 7. FTIR analysis shows the characteristic bands of the oscillation vibrations of the -OH group (3339.90 cm^−1^) and fatty acids (1712.66 cm^−1^). The peak at 1643.02 cm^−1^ confirms the presence of proteins and amides I from the protein structure. A small intensity band in range of 1389–1357 cm^−1^, at 1369.12 cm^−1^, indicates the existence of lipids even after the extraction. Characteristic bands for polysaccharides are confirmed by peaks at 1157.18 cm^−1^ and 1024.94 cm^−1^. Therefore, FT-IR analysis revealed that the method was efficient in obtaining cellulose from *Ulva lactuca* algae [32].

The presence in the extracted cellulose FTIR spectrum of all the peaks characteristic of commercial cellulose indicates that the one obtained is a cellulose of this type.

Table 2 gives the measured swelling degree, established by Equation (1), where m_t_ and m_0_ are the membrane mass at a specific time at the beginning of the swelling. Regarding relationship (1), we demonstrate that, due to the precise determination of membrane mass after swelling, this relationship is frequently used [33,34,35,36,37] to express the degree of swelling, even though it can also be interpreted as membrane absorption capacity.

We can observe that the process of absorption of the alcoholic solution by the membrane occurs rapidly in the first part. After stabilization, the membranes continue to absorb at a slower rate until stabilization or saturation concentration is reached.

(1) $$S_{d}=\frac{m_{t}-m_{0}}{m_{0}}\times 100$$

The low swelling in concentrated ethanol indicates that it is possible to use these membranes for ethanol dehydration of ethanol solutions having the composition similar to the ethanol/water azeotrope.

Numerous mathematical models have been proposed describing the kinetics of hydrogel swelling. In this paper, a model based on the diffusion of the mobile front inflation is proposed.

Empirical, semi-empirical and phenomenological models can be identified in the existing large amount of data and considerations regarding the swelling of polymers (biopolymers). The model proposed in the paper is part of the phenomenological category and has strong elements of novelty and adaptability to other geometric shapes (cylinders and spheres) of polymers that swell. It can also be in the class of models based on diffusion, as discussed in the following, but it differs from them in that fact that it uses as foundation the steady-state diffusion with a variable boundary [38,39,40]. The steady-state diffusion with a variable boundary is defined by a front the kinetic process of structural and vibrational arrangement of polymer fibers takes place.

The Fickian diffusion models apply Fick’s laws to the distribution of solvent in a gel sample during swelling or collapse. These models predict that the fractional approach to equilibrium increases linearly with the square root of time up to roughly 0.4 and that the swelling curve, the fractional approach to equilibrium vs. square root of time, is not sigmoidal even if the diffusion coefficient is a function of composition [30,41,42,43]. Deviations from the fixed boundary Fickian behavior are usually attributed to some of the following phenomena: (i) variable surface concentration, (ii) a history-dependent diffusion coefficient, (iii) stresses between parts of the gel swollen to different extents and (iv) polymer relaxation. The first three have been discussed by Crank et al. [5], while the last has been modeled by Joshi et al. [31,44,45]. Although these models predict the swelling curves for large volume changes reasonably well, they are subject to three objections: (i) they do not allow for the movement of the gel boundary, (ii) they require three or more parameters to fit experimental data and (iii) the diffusion coefficients may show unusual composition dependence, e.g., a maximum at an intermediate composition. However, it has been shown that the sigmoidal swelling behavior can be well described by Fickian diffusion when the movement of the gel surface is considered correctly [32,46].

In this study, we used a diffusion model with a variable effective diffusion coefficient starting from the Fickian model.

**Figure 8:**
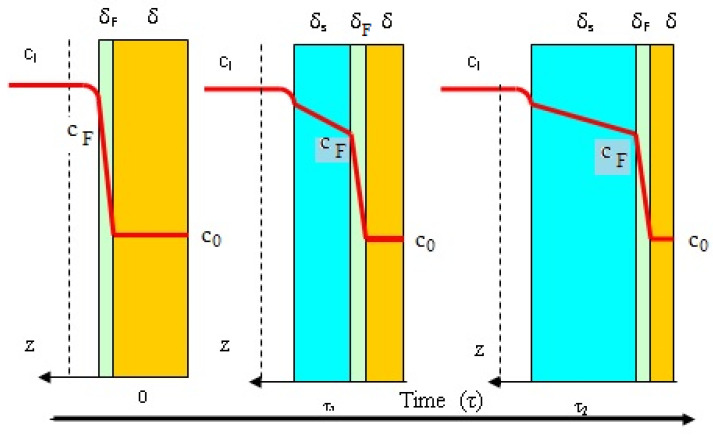
Phenomenological model explaining the dynamics of a membrane swelling (z = 0 in the center of membrane thickness, green: mobile front diffusion, blue: swelled membrane, light brown: non-swelled membrane).

Figure 8 shows a physical model which can explain the swelling of one biopolymer membrane such as pure cellulose and biocellulose or some of those composites between the two. It is observed that the membrane during swelling contains three layers. The external layer of the swelled polymer, where the transported water is the swelling medium, goes by diffusion from saturation concentration to the concentration characterizing the action of the swelling front. The very small intermediate layers, where the kinetic process takes place and the fibrils of biopolymer pass from a pressured to a relative relaxed state, operates with the swelling media difference of concentration C_F_-C_0_. The internal layer is represented by the non-swelled biopolymer. As shown in Figure 8, external layers increase in time, and the intermediate layer keeps its small thickness and disappears at the end of the swelling, whereas the thickness of internal layer decreases during swelling.

According to the presented model for the external layer and for the intermediate layer, the transported swelling medium follows an unsteady-state diffusion process. So, the swelling process can be characterized by the following mathematical model (Equations (2)–(13)).

(2) $$\frac{\partial c}{\partial \tau }=D_{es}\times \frac{\partial^{2}c}{\partial z^{2}}$$

(3) $$\delta +\delta_{F}\leq z\leq \delta +\delta_{F}+\delta_{S},\;\tau =0,\;c=c_{0}$$

(4) $$z=\delta +\delta_{F}+\delta_{S},\;\tau >0,\;c=c_{S}$$

(5) $$z=\delta +\delta_{F},\;\tau >0,\;c=c_{F}$$

(6) $$\frac{\partial c}{\partial \tau }=D_{eF}\times \frac{\partial^{2}c}{\partial z^{2}}$$

(7) $$\delta \leq z\leq \delta +\delta_{F},\;\tau =0,\;c=c_{0}$$

(8) $$z=\delta +\delta_{F},\;\tau >0,\;c=c_{F}$$

(9) $$z=\delta ,\;\tau >0,\;c=c_{0}$$

(10) $$k_{l}\times \left(c_{l}-c_{S}\right)=D_{es}\times {\left(\frac{\partial c}{\partial z}\right)}_{z=\delta +\delta_{F}+\delta_{s}}=D_{es}\times {\left(\frac{\partial c}{\partial z}\right)}_{z=\delta +\delta_{F}}\;=D_{eF}\times {\left(\frac{\partial c}{\partial z}\right)}_{z=\delta +\delta_{F}},\;\tau >0$$

(11) $$c_{F}=c_{\delta +\delta_{F}}$$

(12) $$\frac{\rho_{s}}{M_{s}}\times \frac{{d\delta }_{s}}{d\tau }=\frac{D_{es}}{\delta_{s}}\times \left(c_{S}-c_{F}\right)$$

(13) $$\tau =0,\;\delta_{S}=0$$

(14) $$\frac{\rho_{F}}{M_{s}}\times \frac{{d\delta }_{F}}{d\tau }=\frac{D_{eF}}{\delta_{F}}\times \left(c_{F}-c_{0}\right)$$

(15) $$\tau =0,\;\delta_{F}=0$$

The swelling model, above described, and concentrated through relations (2)–(13) is an unsteady-state diffusion model for movement of swelling media in a membrane in which there is a mobile diffusion front (Figure 8). This 2D model highlights that the diffusion coefficient in the swollen membrane part and the diffusion coefficient in the swelling front are the main model parameters.

The following parts of the model can be clearly distinguished: (a) the unsteady diffusion model for the swelled layer (Equations (2)–(5)), (b) the unsteady diffusion model for the front (intermediate) layer (Equations (8)–(11)), (c) the condition for the localization of swelling media concentration at the surface of the intermediate layer (Equations (9)–(11)) and (d) the dynamics of the swelled and intermediate layers.

We show, without equations, that the above model can be dimensionally reduced to a single diffusion model with a variable effective diffusion coefficient.

The major difficulties of these models are represented by the problem of time dependence of the diffusion frontiers. They can create serious problems for the transposition of numeric models. If the diffusion rate of swelling media is much greater than the rate of moving of swelled interface towards the center of the membrane, then a pseudo-steady state can be assumed. In this case, the above model generates the following diffusion problems (Equations (16)–(18)).

(16) $$\frac{\partial }{\partial z}\left(D_{es}\times \frac{\partial c}{\partial z}\right)=0,\;z=\delta +\delta_{F}+\delta_{s}\to c=c_{s};z=\delta +\delta_{F}\to c=c_{F}$$

(17) $$\frac{\partial }{\partial z}\left(D_{eF}\times \frac{\partial c}{\partial z}\right)=0,\;z=\delta +\delta_{F}\to c=c_{F};z=\delta \to c=c_{0}$$

(18) $${\left(D_{es}\times \frac{\partial c}{\partial z}\right)}_{z=\delta +\delta_{F}}={\left(D_{eF}\times \frac{\partial c}{\partial z}\right)}_{z=\delta +\delta_{F}}$$

Analytic solutions for these problems can be easily obtained. In order to give such a solution, we notice that in the intermediate layer a kinetic process takes place. More precisely, it is expressed by the polymeric fibrils passing from a rigid packed structure to a highly relaxed structure. If we take into consideration the extremely small thickness of this layer, we can note that the kinetic process will occur at its surface. Consequently, the steady-state diffusion model for the intermediate layer is replaced with the expression of surface swelling media flux coupled with the adequate transformation of the relation (Equation (10)). The result is given by the relation (Equation (19)) where the constant of kinetic process of fibrils relaxation, *k_F_*, characterizes the system biopolymer-swelling media, and it is expected to have an important temperature dependence.

(19) $$k_{l}\times \left(c_{l}-c_{s}\right)=\frac{D_{es}}{\delta_{s}}\times \left(c_{s}-c_{F}\right)=k_{F}\times \left(c_{F}-c_{0}\right)$$

The coupling of the relation of steady-state diffusion in the swelling layer (Equation (16)) with the relation (Equation (19)) allows us to write the expressions of concentrations *c_S_* and *c_F_* depending on the swelling layer thickness (Equations (19) and (20)).

(20) $$c_{s}\left(\delta_{s}\right)=\frac{k_{l}\times c_{l}+\frac{D_{es}}{\delta_{s}}\times k_{F}\times \frac{c_{0}}{k_{F}+\frac{D_{es}}{\delta_{s}}}}{\frac{D_{es}}{\delta_{s}}+k_{l}-\frac{D_{es}}{\delta_{s}}\times \left(\frac{\frac{D_{es}}{\delta_{s}}}{k_{F}+\frac{D_{es}}{\delta_{s}}}\right)}$$

(21) $$c_{F}\left(\delta_{s}\right)=\frac{\frac{D_{es}}{\delta_{s}}\times c_{s}\left(\delta_{s}\right)+k_{F}\times c_{0}}{\frac{D_{es}}{\delta_{s}}+k_{F}}$$

With respect to $c_{s}\left(\delta_{s}\right)$, it is shown that if the values go over equilibrium concentration (liquid membrane concentration when the saturation is finished), then $c_{s}\;(\delta_{s})$ takes this value.

In order to obtain the swelling dynamics model, relations (Equation (20)) and (Equation (21)) are connected to the relation (Equation (11)), transcribed below so as to emphasize the dynamics of the thickness of the swelling layer.

(22) $$\frac{d\delta_{s}}{d\tau }=\frac{D_{es}}{\delta_{s}}\times \frac{M_{s}}{\rho_{s}}\times \left(c_{s}\left(\delta_{s}\right)-c_{F}\left(\delta_{s}\right)\right)$$

The mathematical model expressed by the assembly of relations (Equations (20)–(22)) cannot be integrated with the initial relation (Equation (13)). In order to solve this small problem, we consider that at the beginning of swelling process, on the membrane surface, a kinetic process takes place which shows the passing of the biopolymer fibrils from a rigid to a relaxed structure (see the schema from Figure 8 for *τ* = 0). The phenomenological analysis of this consideration leads to a solution which permits the expression of the initial condition associated with a differential equation, showing the increase in the swelling layers (Equation (22)). This is also highlighted by the relation (Equation (23)) where Δ*τ* is the time interval for the appearance of the swelled structure on the membrane surface.

(23) $$\tau =0,\;\delta_{s0}=\frac{k_{F}\times M_{sm}}{\rho_{m}\times \left(k_{F}+k_{l}\right)}\times k_{l}\times \left(c_{l}-c_{0}\right)\times \Delta \tau$$

To obtain the final model of biopolymer swelling, the assembly of relations (Equations (20)–(23)) is completed with the balance of polymeric material and with the expression of the swelling ratio. On this basis, the differential equation (Equation (24)) and the relation (Equation (26)) are derived. The expression of the actual degree of swelling of the membrane described by relation (26) agrees with the expression in relation (1), which is used to capitalize on experimental results. The model developed this way has the ability to express the swelling of the membrane as an increase in volume.

(24) $$\frac{d\delta }{d\tau }=\frac{\rho_{s}-M_{sm}\times c_{s}\left(\delta_{s}\right)}{\rho_{0}-M_{sm}\times c_{0}}\times \frac{d\delta_{s}}{d\tau }$$

(25) $$\tau =0,\;\delta =\delta_{0}$$

(26) $$S_{\tau }=\frac{\delta_{s}\times \rho_{s}}{\delta_{0}\times \rho_{0}}$$

In the above relation, the density of the swelled layer can be appreciated as shown in the relation (Equation (27)) where ρ_0_ is the density of the non-swelled polymer.

(27) $$\rho_{s}=\frac{\delta_{s}}{\delta_{s}+\delta }\times \rho_{sm}+\frac{\delta }{\delta_{s}+\delta }\times \rho_{0}$$

**Figure 9:**
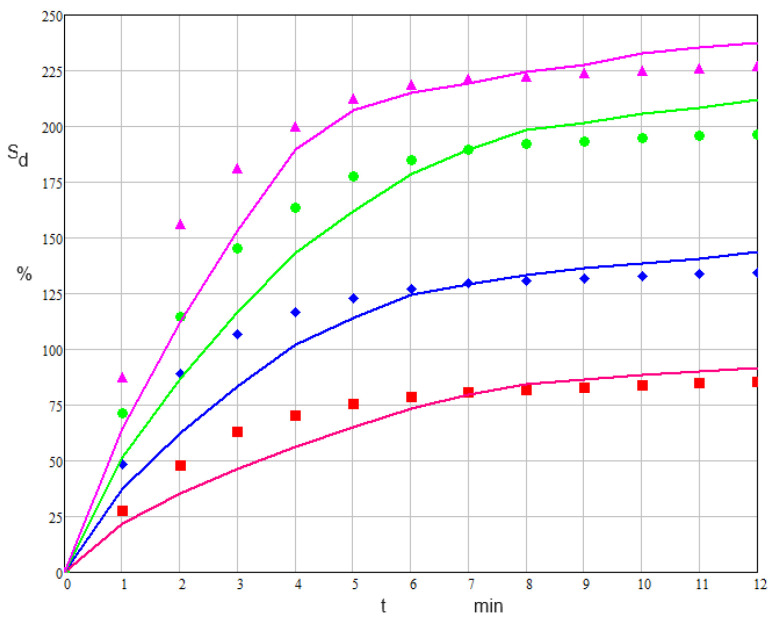
Dynamics of *Ulva lactuca* cellulose swelling degree of the model and experiment (line (model) red: 99% ethanol, blue: 80% ethanol, green: 60% ethanol, magenta: 40% ethanol; points (experimental) red: 99% ethanol, blue: 80% ethanol, green: 60% ethanol, magenta: 40% ethanol).

The developed model thus has as its main parameter the diffusion coefficient of the liquid phase through the swelled polymer structure. The characteristic kinetic constant showing the structural change in the diffusion front appears as a secondary parameter in the model. It must be considered that the diffusion front is finally embedded in the swollen polymer. The testing of the model on biocellulose swelling showed that we can express the current *k_F_* as in the relation (Equation (28)), where *k_F_*_0_ shows the specificity of the polymers and of the swelling liquids. Using experimental swelling data from Table 2, we can identify the best values of *D_es_* and $k_{F0}$ by minimizing the function which expresses the mean squared deviation between these data and those calculated according to the model. The identified *D_es_* and $k_{F0}$, given by Table 3, show that these parameters are strongly influenced by the water content of the swelling medium (Figure 9).

(28) $$k_{F}=k_{F0}{\left(\frac{\delta_{s}}{\delta +\delta_{s}}\right)}^{0.5}$$

## 4. Conclusions

This study proposed and implemented a procedure for extracting cellulose from the algal species *Ulva lactuca* that is found in abundance along the Romanian Black Sea Coastline. A physicochemical characterization of the algal powder *Ulva lactuca* was conducted, but also of the biocellulose obtained from the algal material, using optic microscopy, SEM and FTIR. The extraction of biocellulose was performed through ethanol precipitation, which includes the removal of lipids, pigments, ulvans and hemicellulose without affecting the cellulose obtained as a white powder. The highest yield of extracted cellulose (20,944%) was obtained for the following factors (S/L = 1/20; conc. ethanol = 90%, conc. salts = 4 g/L), this proving that *Ulva lactuca* is a viable alternative resource in cellulose production.

Also, a procedure was performed to obtain membranes from cellulose extracted from the algal species *Ulva lactuca*. The swelling of biocellulose membranes in alcoholic solutions of high concentrations was investigated. It can be observed that the process of absorption of the alcoholic solution by the membrane occurs rapidly in the first part. After stabilization, the membranes continue to absorb at a slower rate until the stabilization or saturation concentration is reached.

The low swelling in concentrated ethanol indicates that it is possible to use these membranes for ethanol dehydration of ethanol solutions with a composition similar to the ethanol/water azeotrope.

The developed mathematical model thus has as its main parameter the diffusion coefficient of the liquid phase through the swelled polymer structure. The characteristic kinetic constant showing the structural change in the diffusion front appears as a secondary parameter in the model. It must be considered that the diffusion front is finally embedded in the swollen polymer. The identified *D_es_* (diffusion rate of swelling media) and $k_{F0}$ (which characterizes the system biopolymer-swelling media) shows that these parameters are extremely strongly influenced by the water content of the swelling medium according to the mathematical model. The purpose of studying and developing membranes is to use them in the processes of separation of alcohol/water mixtures for solvent recovery and reuse.

## Figures and Tables

**Table 1:** Experimental determinations regarding the extraction of cellulose from algae.

No. crt.	Solid/Liquid Ratio, S	Ethanol Concentration %, E	Concentration of Salts %, C		Cellulose Yield, R
			g/L	Ppm	
1	1/20	90%	2	0.085	20.817% ± 0.004
2	1/20	90%	4	0.898	20.944% ± 0.002
3	1/20	60%	2	0.085	4.494% ± 0.001
4	1/20	60%	4	0.898	4.625% ± 0.007
5	1/10	90%	2	0.085	15.022% ± 0.005
6	1/10	90%	4	0.898	15.829% ± 0.003
7	1/10	60%	2	0.085	3.781% ± 0.004
8	1/10	60%	4	0.898	4.030% ± 0.006

**Table 2:** Dynamics of *Ulva lactuca* cellulose swelling degree (t = 20 °C, $\delta_{0}$ = 2.5 mm).

	t min	0	1	2	3	4	5	6	7	8	9	10	11	12
Sd %	Eth 99%	0	27.5	47.9	63.2	70.4	75.5	78.6	80.7	81.4	82.3	83.5	84.4	85.1
	Eth 80%	0	48.2	89.1	106.9	122.8	126.7	129.5	130.4	131.8	132.7	133.4	133.9	134.4
	Eth 60%	0	71.2	114.3	145.5	163.4	177.8	185.1	189.8	192.2	193.4	194.6	195.8	196.1
	Eth 40%	0	87.5	156.2	181.2	200.1	212.5	218.7	221.2	222.6	223.7	225.1	226.2	226.8

**Table 3:** Identified values of model parameters swelling of *Ulva lactuca* cellulose membrane.

N.c	Eth. Conc (%)	*k_F_*_0_ (m/s)	*D_es_* m^2^/s
1	99	4.5 × 10^−3^	0.08 × 10^−10^
2	80	7.3 × 10^−3^	0.25 × 10^−10^
3	60	9.7 × 10^−3^	0.52 × 10^−10^
4	40	11.5 × 10^−3^	1.13 × 10^−10^

## Data Availability

Data are contained within the article.
